# Supplementary material for: Comparative genomic analysis of a metagenome-assembled genome reveals distinctive symbiotic traits in a Mucoromycotina fine root endophyte arbuscular mycorrhizal fungus
Source: BMC Genomics. 2025 Oct 29;26:967. doi: 10.1186/s12864-025-12149-w (PMC12574304; doi:10.1186/s12864-025-12149-w)
Supplement: Supplementary file 3 — Supplementary Material 3. [file 12864_2025_12149_MOESM3_ESM.pptx]

## Slide 1
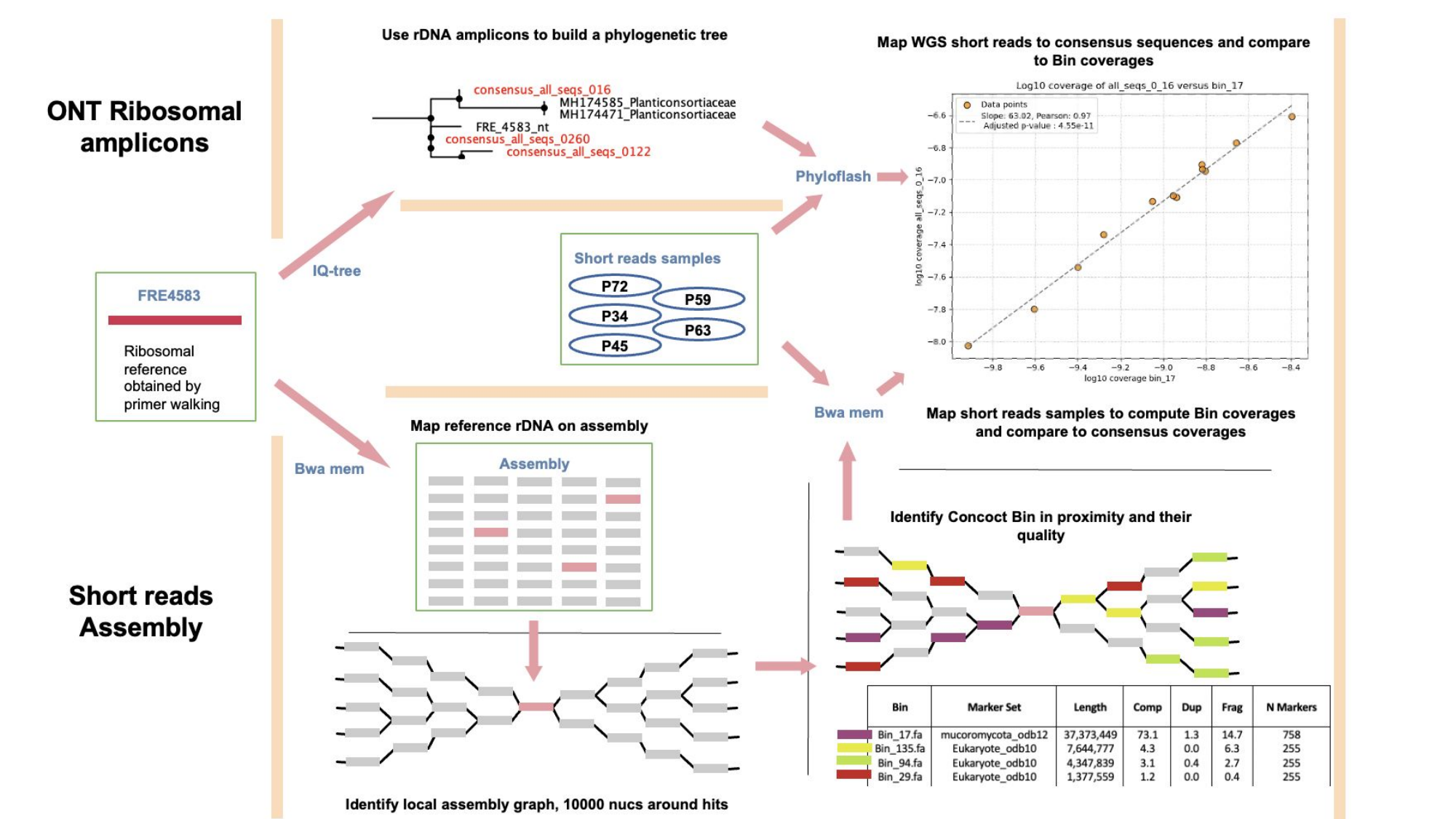

Figure 1. Bioinformatic workflow leading to identification of the FRE MAG: FRE4583 is a reference sequence obtained by primer walking, it is used, to 1) identify similar unitigs in the assembly graph and 2) identify a clade of de novo ont ribosomal amplicon. As a part of the treatment of the assembly, the contigs are binned using CONCOCT and we focus on the few which are found to be in proximity of the flagged unitig. The coverage of Bin_17, the only bin of good quality, is then compared to the coverage of the multiple ont amplicon from the FRE clade. Consensus_all_seqs_016 is perfectly correlated with Bin_17, validating our finding.

## Slide 2
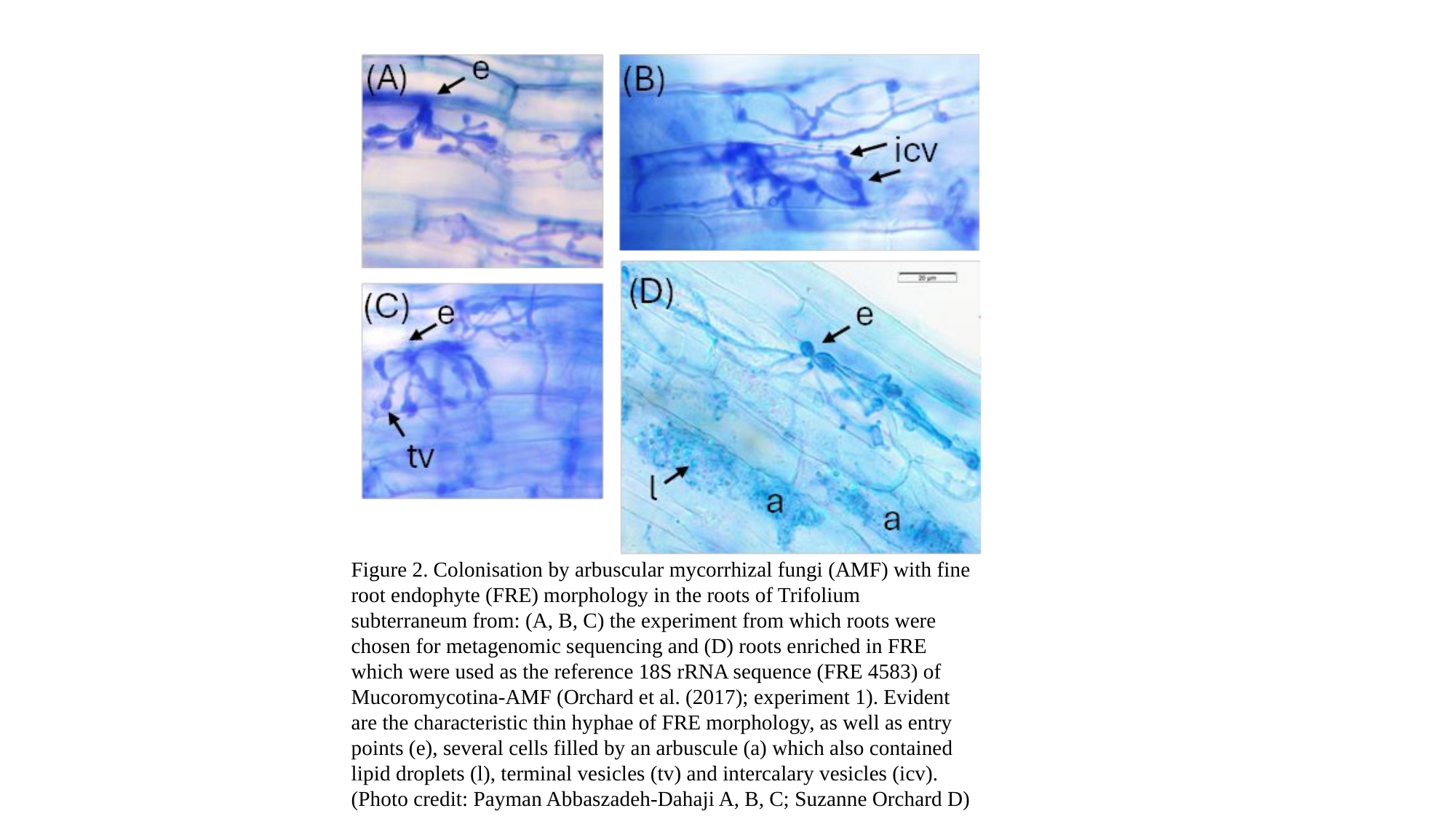

Figure 2. Colonisation by arbuscular mycorrhizal fungi (AMF) with fine root endophyte (FRE) morphology in the roots of Trifolium subterraneum from: (A, B, C) the experiment from which roots were chosen for metagenomic sequencing and (D) roots enriched in FRE which were used as the reference 18S rRNA sequence (FRE 4583) of Mucoromycotina-AMF (Orchard et al. (2017); experiment 1). Evident are the characteristic thin hyphae of FRE morphology, as well as entry points (e), several cells filled by an arbuscule (a) which also contained lipid droplets (l), terminal vesicles (tv) and intercalary vesicles (icv). (Photo credit: Payman Abbaszadeh-Dahaji A, B, C; Suzanne Orchard D)

## Slide 3
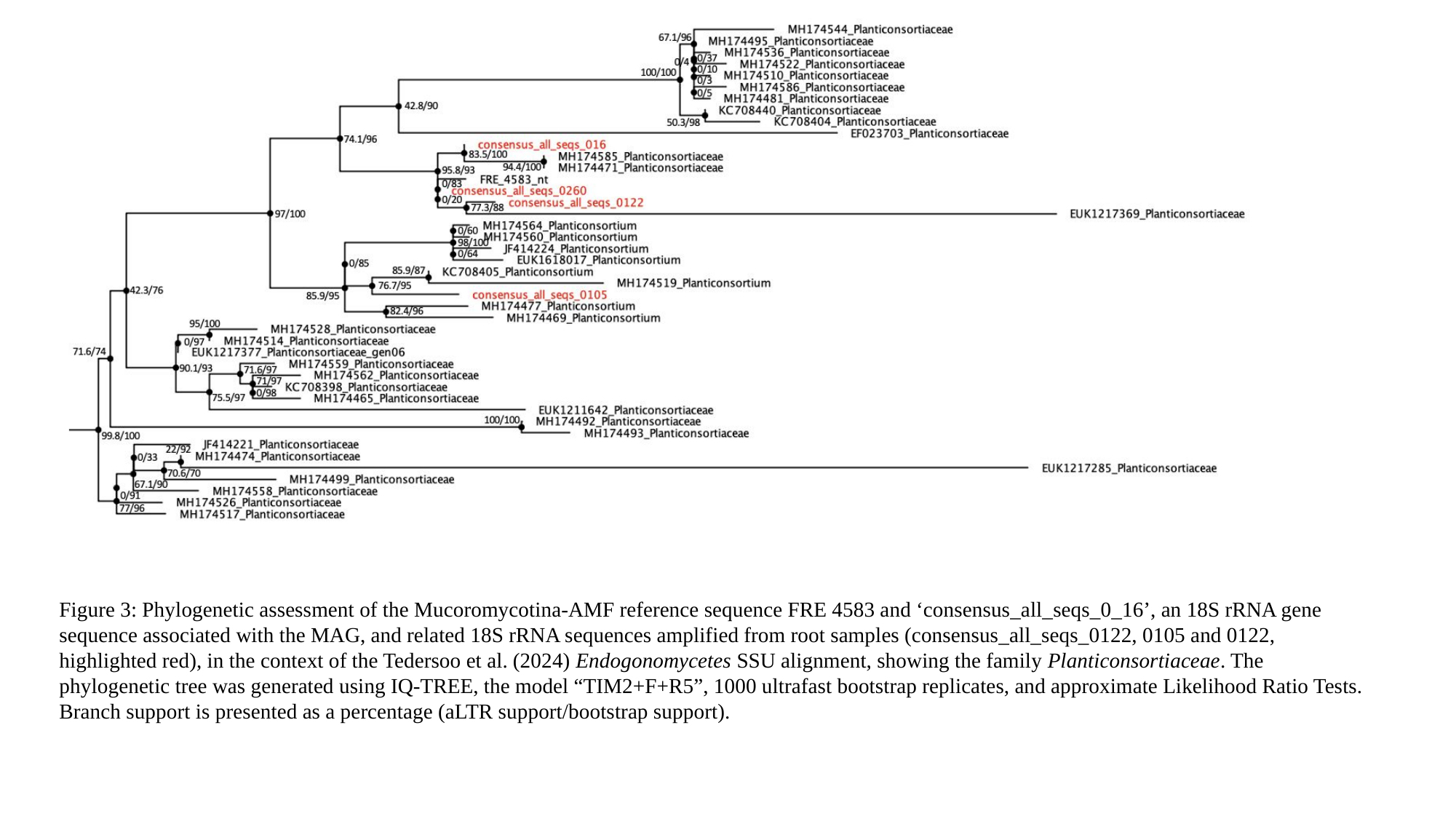

Figure 3: Phylogenetic assessment of the Mucoromycotina-AMF reference sequence FRE 4583 and ‘consensus_all_seqs_0_16’, an 18S rRNA gene sequence associated with the MAG, and related 18S rRNA sequences amplified from root samples (consensus_all_seqs_0122, 0105 and 0122, highlighted red), in the context of the Tedersoo et al. (2024) Endogonomycetes SSU alignment, showing the family Planticonsortiaceae. The phylogenetic tree was generated using IQ-TREE, the model “TIM2+F+R5”, 1000 ultrafast bootstrap replicates, and approximate Likelihood Ratio Tests. Branch support is presented as a percentage (aLTR support/bootstrap support).

## Slide 4
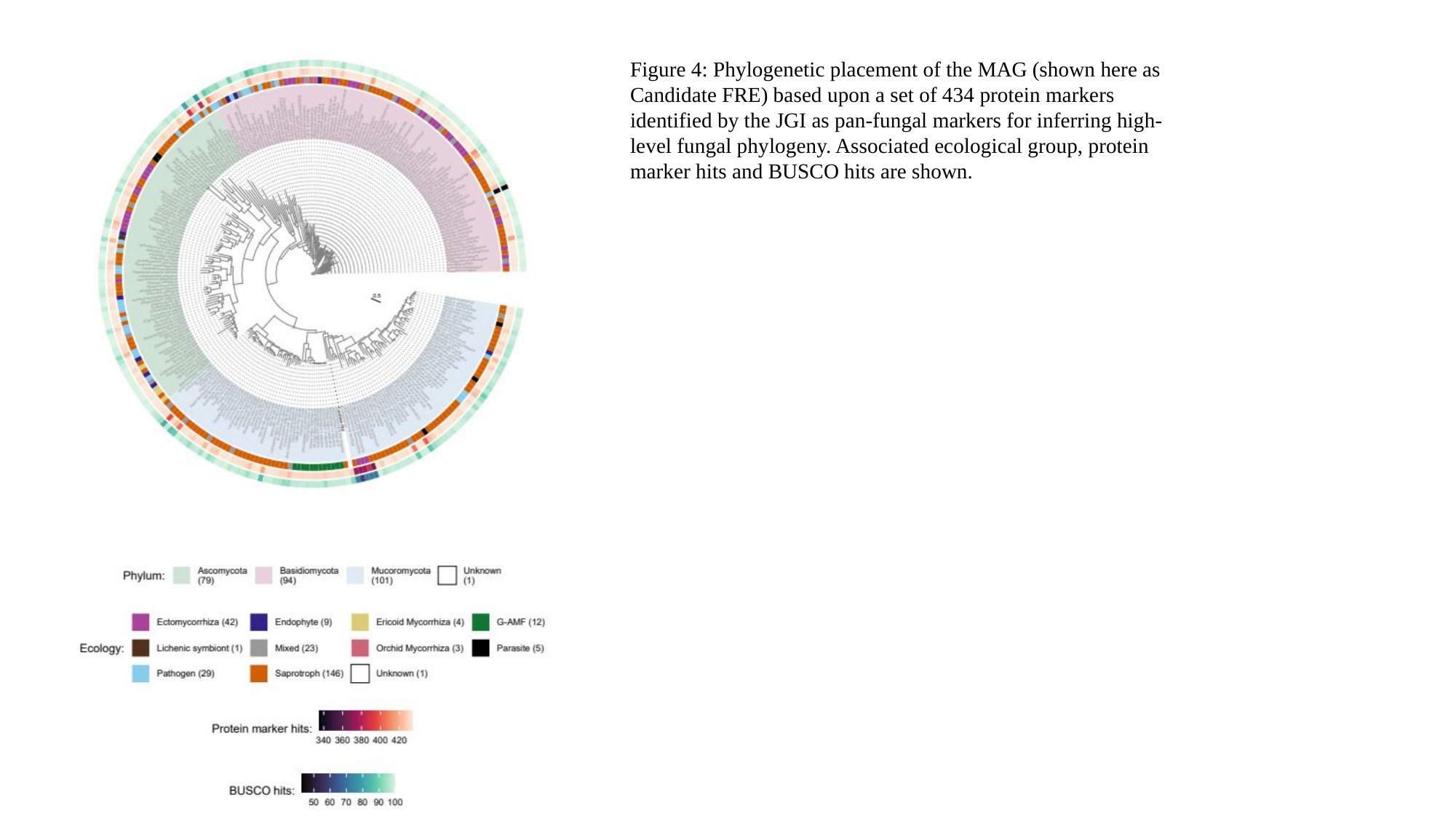

Figure 4: Phylogenetic placement of the MAG (shown here as Candidate FRE) based upon a set of 434 protein markers identified by the JGI as pan-fungal markers for inferring high-level fungal phylogeny. Associated ecological group, protein marker hits and BUSCO hits are shown.

## Slide 5
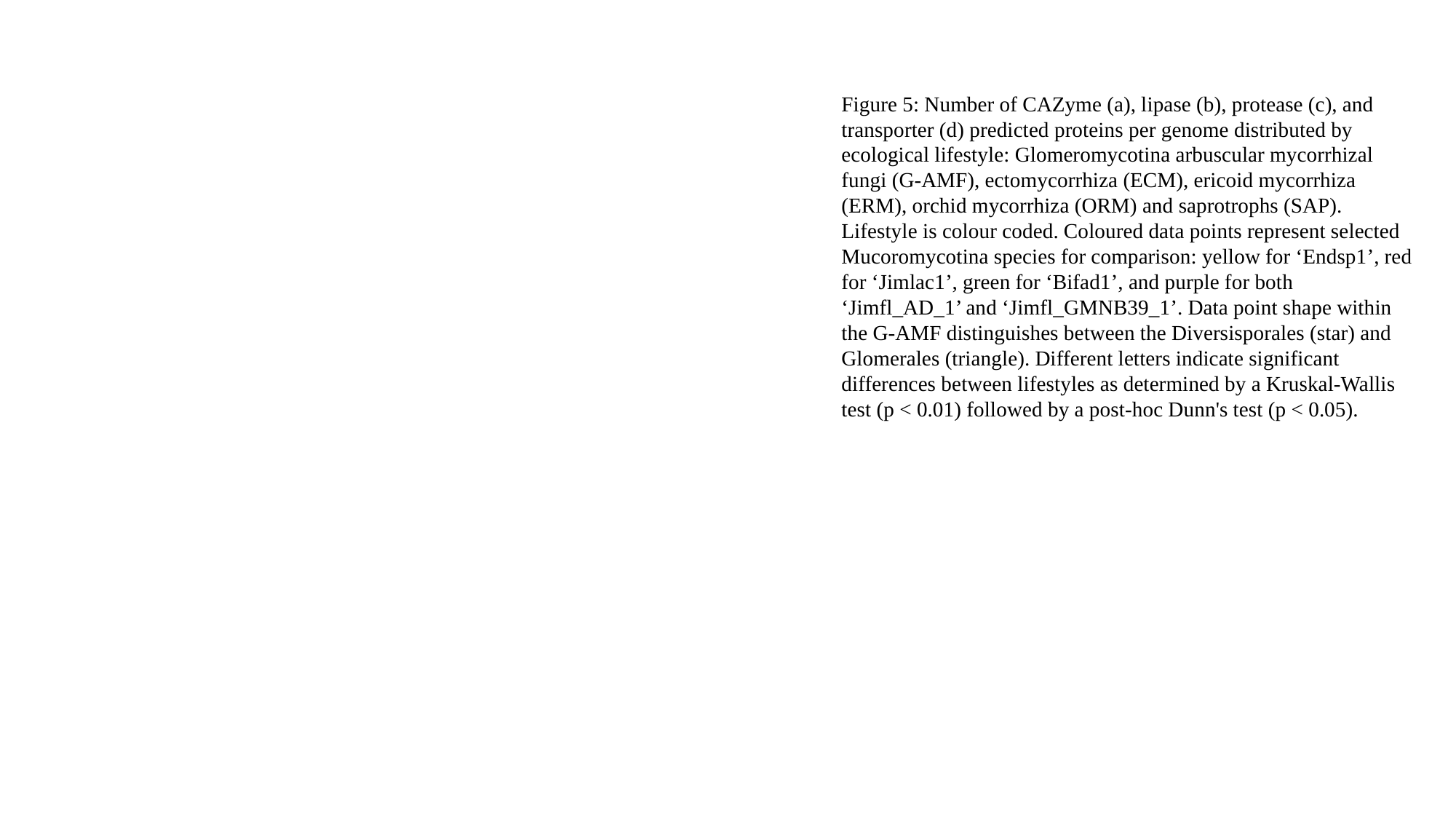

Figure 5: Number of CAZyme (a), lipase (b), protease (c), and transporter (d) predicted proteins per genome distributed by ecological lifestyle: Glomeromycotina arbuscular mycorrhizal fungi (G-AMF), ectomycorrhiza (ECM), ericoid mycorrhiza (ERM), orchid mycorrhiza (ORM) and saprotrophs (SAP). Lifestyle is colour coded. Coloured data points represent selected Mucoromycotina species for comparison: yellow for ‘Endsp1’, red for ‘Jimlac1’, green for ‘Bifad1’, and purple for both ‘Jimfl_AD_1’ and ‘Jimfl_GMNB39_1’. Data point shape within the G-AMF distinguishes between the Diversisporales (star) and Glomerales (triangle). Different letters indicate significant differences between lifestyles as determined by a Kruskal-Wallis test (p < 0.01) followed by a post-hoc Dunn's test (p < 0.05).

## Slide 6
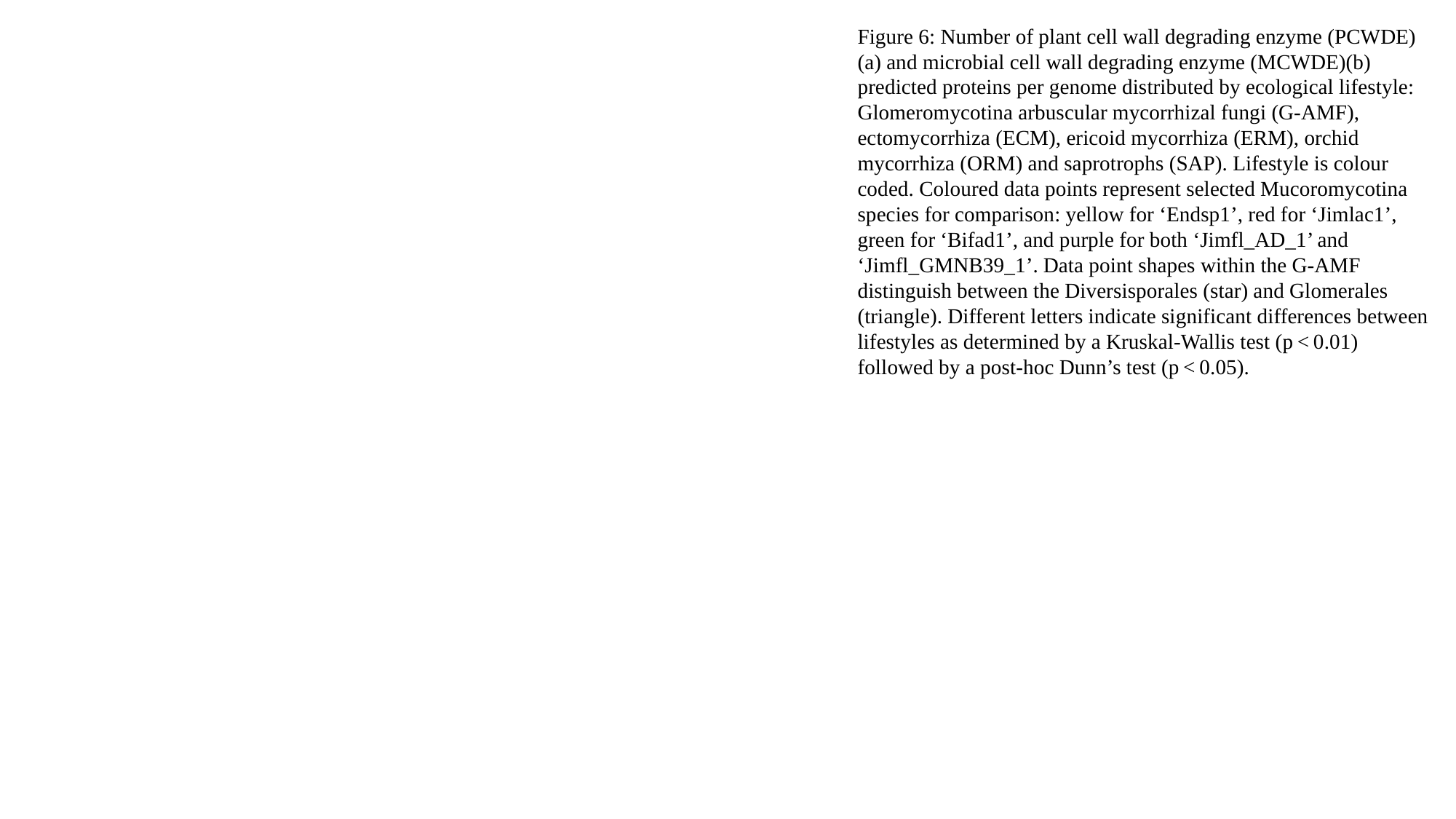

Figure 6: Number of plant cell wall degrading enzyme (PCWDE)(a) and microbial cell wall degrading enzyme (MCWDE)(b) predicted proteins per genome distributed by ecological lifestyle: Glomeromycotina arbuscular mycorrhizal fungi (G-AMF), ectomycorrhiza (ECM), ericoid mycorrhiza (ERM), orchid mycorrhiza (ORM) and saprotrophs (SAP). Lifestyle is colour coded. Coloured data points represent selected Mucoromycotina species for comparison: yellow for ‘Endsp1’, red for ‘Jimlac1’, green for ‘Bifad1’, and purple for both ‘Jimfl_AD_1’ and ‘Jimfl_GMNB39_1’. Data point shapes within the G-AMF distinguish between the Diversisporales (star) and Glomerales (triangle). Different letters indicate significant differences between lifestyles as determined by a Kruskal-Wallis test (p < 0.01) followed by a post-hoc Dunn’s test (p < 0.05).

## Slide 7
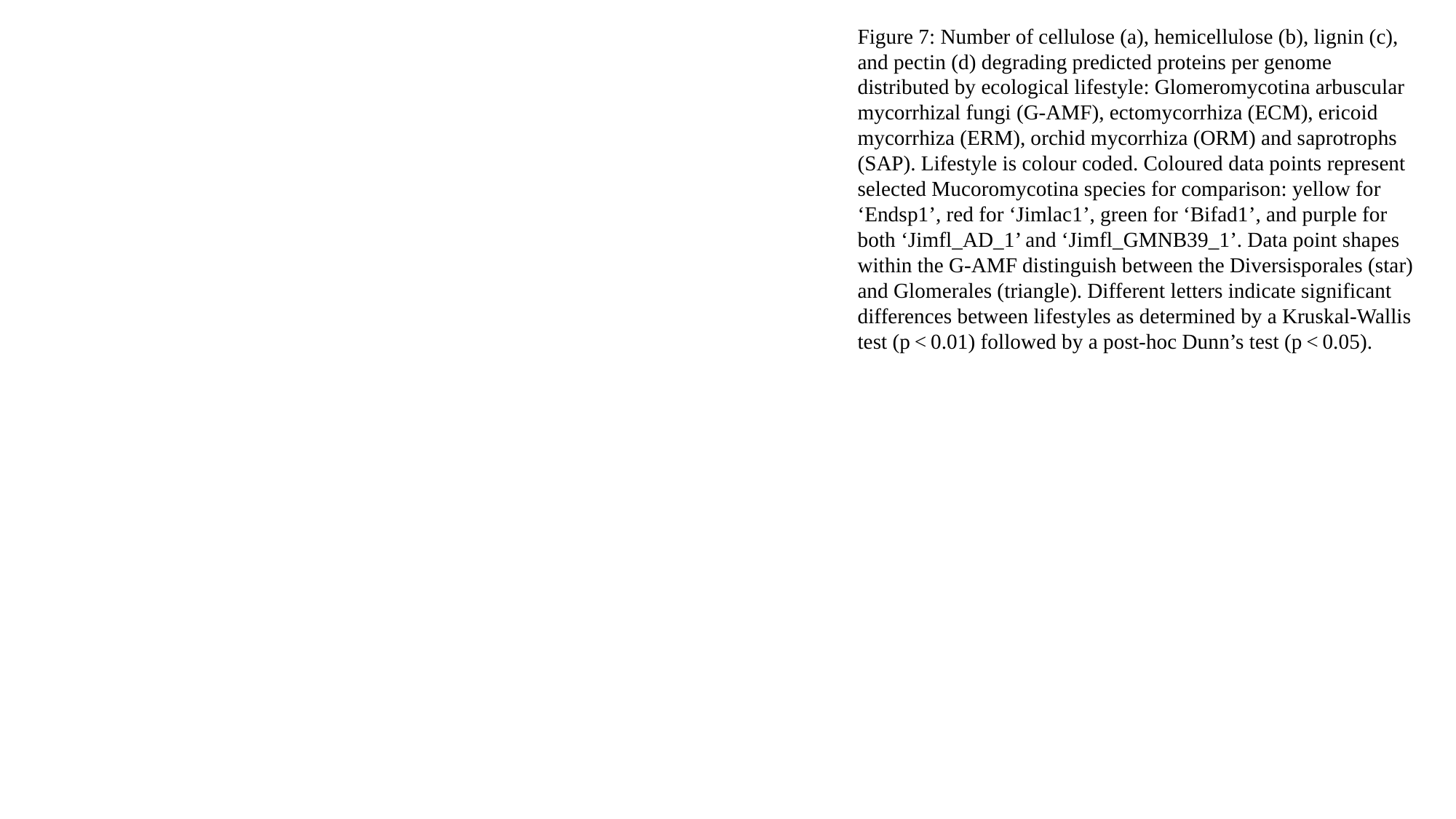

Figure 7: Number of cellulose (a), hemicellulose (b), lignin (c), and pectin (d) degrading predicted proteins per genome distributed by ecological lifestyle: Glomeromycotina arbuscular mycorrhizal fungi (G-AMF), ectomycorrhiza (ECM), ericoid mycorrhiza (ERM), orchid mycorrhiza (ORM) and saprotrophs (SAP). Lifestyle is colour coded. Coloured data points represent selected Mucoromycotina species for comparison: yellow for ‘Endsp1’, red for ‘Jimlac1’, green for ‘Bifad1’, and purple for both ‘Jimfl_AD_1’ and ‘Jimfl_GMNB39_1’. Data point shapes within the G-AMF distinguish between the Diversisporales (star) and Glomerales (triangle). Different letters indicate significant differences between lifestyles as determined by a Kruskal-Wallis test (p < 0.01) followed by a post-hoc Dunn’s test (p < 0.05).

## Slide 8
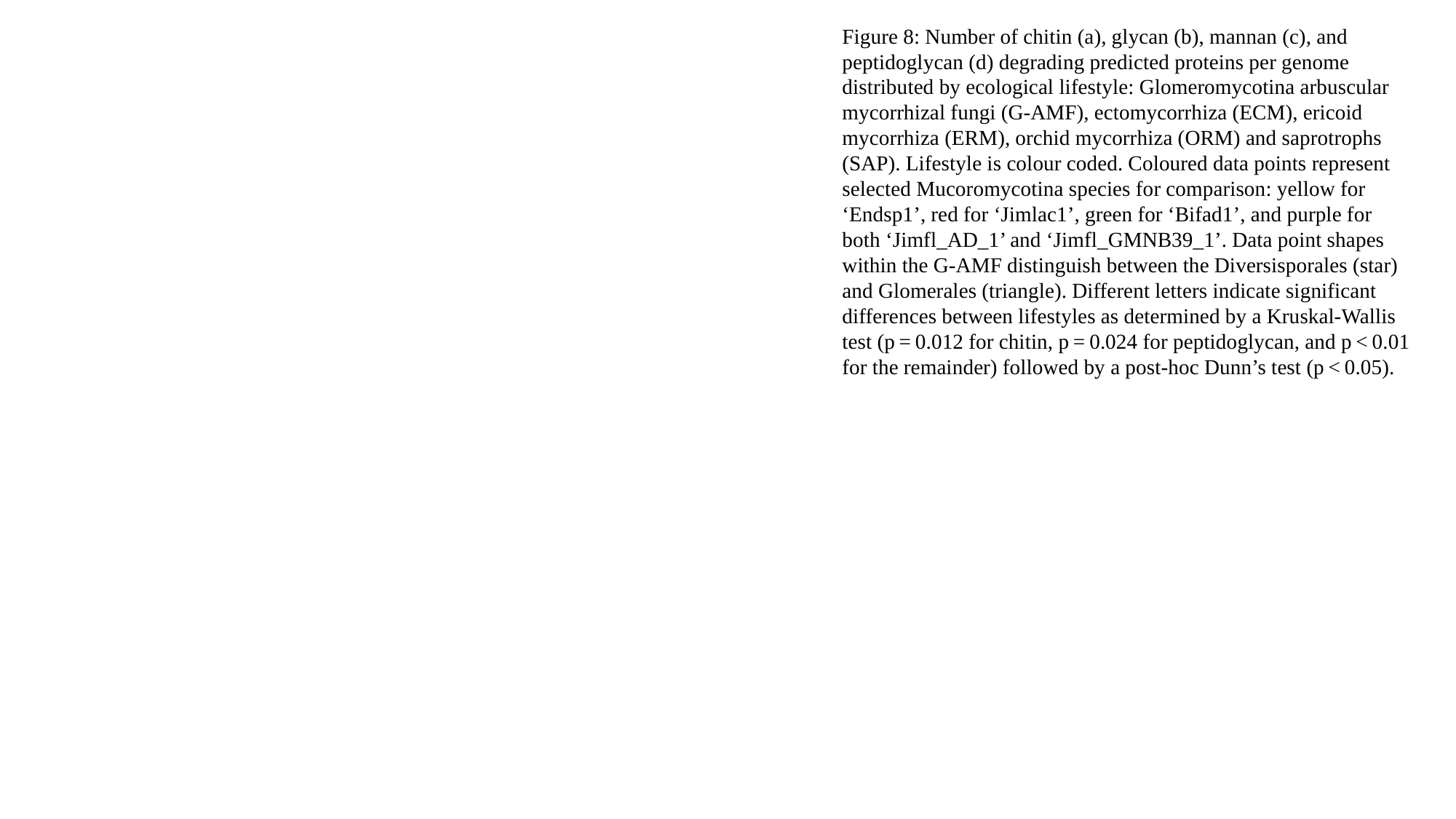

Figure 8: Number of chitin (a), glycan (b), mannan (c), and peptidoglycan (d) degrading predicted proteins per genome distributed by ecological lifestyle: Glomeromycotina arbuscular mycorrhizal fungi (G-AMF), ectomycorrhiza (ECM), ericoid mycorrhiza (ERM), orchid mycorrhiza (ORM) and saprotrophs (SAP). Lifestyle is colour coded. Coloured data points represent selected Mucoromycotina species for comparison: yellow for ‘Endsp1’, red for ‘Jimlac1’, green for ‘Bifad1’, and purple for both ‘Jimfl_AD_1’ and ‘Jimfl_GMNB39_1’. Data point shapes within the G-AMF distinguish between the Diversisporales (star) and Glomerales (triangle). Different letters indicate significant differences between lifestyles as determined by a Kruskal-Wallis test (p = 0.012 for chitin, p = 0.024 for peptidoglycan, and p < 0.01 for the remainder) followed by a post-hoc Dunn’s test (p < 0.05).

## Slide 9
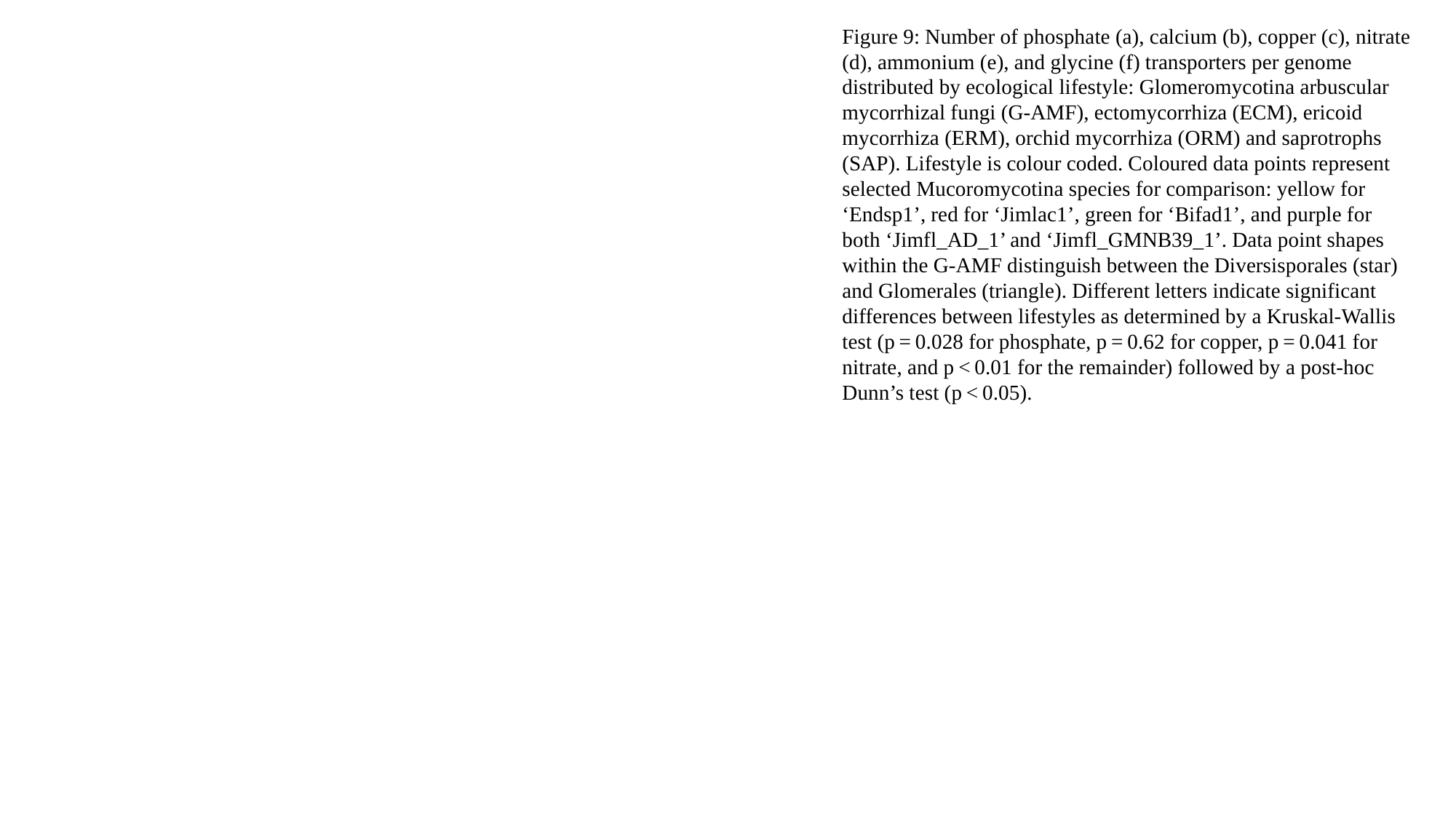

Figure 9: Number of phosphate (a), calcium (b), copper (c), nitrate (d), ammonium (e), and glycine (f) transporters per genome distributed by ecological lifestyle: Glomeromycotina arbuscular mycorrhizal fungi (G-AMF), ectomycorrhiza (ECM), ericoid mycorrhiza (ERM), orchid mycorrhiza (ORM) and saprotrophs (SAP). Lifestyle is colour coded. Coloured data points represent selected Mucoromycotina species for comparison: yellow for ‘Endsp1’, red for ‘Jimlac1’, green for ‘Bifad1’, and purple for both ‘Jimfl_AD_1’ and ‘Jimfl_GMNB39_1’. Data point shapes within the G-AMF distinguish between the Diversisporales (star) and Glomerales (triangle). Different letters indicate significant differences between lifestyles as determined by a Kruskal-Wallis test (p = 0.028 for phosphate, p = 0.62 for copper, p = 0.041 for nitrate, and p < 0.01 for the remainder) followed by a post-hoc Dunn’s test (p < 0.05).
